# Shaping the future of preclinical development of successful disease-modifying drugs against Alzheimer's disease: a systematic review of tau propagation models

**DOI:** 10.1186/s40478-024-01748-5

**Published:** 2024-04-04

**Authors:** Neha Basheer, Luc Buee, Jean-Pierre Brion, Tomas Smolek, Muhammad Khalid Muhammadi, Jozef Hritz, Tomas Hromadka, Ilse Dewachter, Susanne Wegmann, Isabelle Landrieu, Petr Novak, Amritpal Mudher, Norbert Zilka

**Affiliations:** 1 Institute of Neuroimmunology, Slovak Academy of Sciences, Dubravska Cesta 9, 845 10 Bratislava, Slovakia; 2 Inserm, CHU Lille, CNRS, LilNCog - Lille Neuroscience & Cognition, University of Lille, 59000 Lille, France; 3 Faculty of Medicine, Laboratory of Histology, Alzheimer and Other Tauopathies Research Group (CP 620), ULB Neuroscience Institute (UNI), Université Libre de Bruxelles, 808, Route de Lennik, 1070 Brussels, Belgium; 4 CEITEC Masaryk University, Kamenice 5, 625 00 Brno, Czech Republic; 5 Department of Chemistry, Faculty of Science, Masaryk University, Kamenice 5, 62500 Brno, Czech Republic; 6 Biomedical Research Institute, BIOMED, Hasselt University, 3500 Hasselt, Belgium; 7 German Center for Neurodegenerative Diseases, Charitéplatz 1, 10117 Berlin, Germany; 8 Einstein Center for Neurosciences Berlin, Charité - Universitätsmedizin Berlin, Berlin, Germany; 9 CNRS EMR9002 - BSI - Integrative Structural Biology, 59000 Lille, France; 10 Inserm, CHU Lille, Institut Pasteur de Lille, U1167 - RID-AGE - Risk Factors and Molecular Determinants of Aging-Related Diseases, University of Lille, 59000 Lille, France; 11 School of Biological Sciences, Faculty of Environment and Life Sciences, University of Southampton, Highfield Campus, Southampton, SO17 1BJ UK; 12 AXON Neuroscience R&D Services SE, Dubravska Cesta 9, 845 10 Bratislava, Slovakia

**Keywords:** Tau protein, Propagation, Spreading, Aggregation, Neurofibrillary tangles, Animal models

## Abstract

The transcellular propagation of the aberrantly modified protein tau along the functional brain network is a key hallmark of Alzheimer's disease and related tauopathies. Inoculation-based tau propagation models can recapitulate the stereotypical spread of tau and reproduce various types of tau inclusions linked to specific tauopathy, albeit with varying degrees of fidelity. With this systematic review, we underscore the significance of judicious selection and meticulous functional, biochemical, and biophysical characterization of various tau inocula. Furthermore, we highlight the necessity of choosing suitable animal models and inoculation sites, along with the critical need for validation of fibrillary pathology using confirmatory staining, to accurately recapitulate disease-specific inclusions. As a practical guide, we put forth a framework for establishing a benchmark of inoculation-based tau propagation models that holds promise for use in preclinical testing of disease-modifying drugs.

## Introduction

Alzheimer’s disease (AD) and related tauopathies are characterized by the propagation of fibrillary aggregates primarily composed of pathologically altered tau protein. Through extensive biochemical and neuropathological studies [21, 22, 37, 113, 119] as well as corroborative evidence from tau positron emission tomography (tau-PET) [50, 151], a hierarchical and stereotypical pattern of fibrillary tau deposition has been confirmed. Importantly, tau deposition in the brain consistently correlates with clinical symptoms and progression across all forms of tauopathies [53, 54]. This underscores the importance of targeting tau propagation to effectively intervene in and decelerate the progression of tauopathies.

Pathologically altered tau exhibits conformational changes leading to the assembly of fibrillary tau aggregates. These aggregates can break into smaller pieces or serve as a surface for secondary nucleation, resulting in further aggregation along interconnected neuronal networks in a stereotypical manner [61, 83, 112]. Traditional transgenic models leverage diverse promoters, regulatory elements, and inducible systems to enhance the faithful recapitulation of tau pathology and propagation. Nonetheless, these models exhibit constraints in the exploration of tau propagation dynamics, as the transgenic expression of pathological tau tends to induce local replication rather than distal spread. Conversely, models characterized by localized tau expression, for instance rTgTauEC [36], warrant consideration. However, a notable challenge emerges in striving to faithfully recapitulate disease-specific tau inclusions and attain a more substantial tangle load, which is essential for visualizing the effect size of any disease-modifying therapy (DMT).

In the framework of this review, we will exclusively focus on inoculation-based tau propagation models that entail the intracerebral tau administration in wild-type (WT) or tau transgenic (Tg) rodent models. Where the inoculum can be derived from either human or rodent brains or synthetically generated as preformed tau fibrils (PFFs). These models can effectively recapitulate tau propagation and disease-specific tau inclusions, thereby proving instrumental in overcoming the limitations associated with traditional transgenic models [108]. This approach not only presents a promising avenue for overcoming limitations, providing models for mechanistic study and for the study of downstream processes, but also offers an enticing opportunity for evaluating the efficacy of therapeutic interventions.

In the subsequent sections, we will engage in a thorough discussion covering the spectrum of tau-inoculum choices and their characterization, as well as the methods for characterizing and validating resultant tau pathology, exploring regional brain vulnerability, and carefully selecting appropriate animal models for these purposes. The overarching aim is to underscore the utility of these models for future preclinical efficacy studies, providing insights through a review of pertinent studies employing these models.

## Methods

### Search strategy

A comprehensive literature retrieval was performed to identify original studies investigating tau propagation upon intracerebral inoculation of various tau inocula in Tg/WT mice. We gathered the pertinent literature from four independent databases: PubMed, Scopus, Web of Science, and Google Scholar. For the literature search, we used “tau” AND “spreading” OR “propagation” AND “injections” OR “seeding” OR “inoculation” AND “animal models” OR “mouse” OR “rat” as the keywords. The search was restricted to the English language with no restrictions on the date of publication. In Google Scholar, only those articles whose titles included the keywords were selected, to achieve a fairly accurate retrieval. We evaluated the qualifications of the animal studies independently according to the inclusion criteria by screening the abstracts and methodology sections of the identified publications.

### Inclusion criteria

The inclusion criteria for in vivo modelling of tau propagation were as follows:

1. Original experimental studies.
2. *Types of animals:* Wild-type (WT) or tau transgenic (Tg) rodent models of any age, sex, or strain.
3. *Types of studies:* To be included in the review, the study must contain at least one tau inoculation group where induction of pathology was observed.
4. *Types of outcomes evaluated:* Any methodological studies that investigated the induction of tau propagation by human or rodent brain-derived free oligomeric or fibrillar tau, or in the encapsulated form in exosomes. Alternatively, studies involving synthetic preformed fibrils (PFFs) were also included.

### Exclusion criteria

1. Duplicated references;
2. Review articles; abstracts; letters; comments;
3. Literature with incomplete data or propagation not assessed.

### Data extraction and quality assessment

The following information from each study is listed in Table 1, 2, and 3:

1. The experimental model, including the name, type of expressed tau, any other modification, and strain
2. Age at inoculation
3. Age at termination
4. Type and amount of inoculum
5. Brain region of isolation
6. The coordinates of the injection
7. Speed of application
8. Characteristics of the inclusions
9. Regions with observed tau propagation

**Table 1:** Summary of studies involving human brain-derived tau inoculation

Experimental animal;age at inoculation	Time of termination	Pathological tau (mass; volume)	Brain region of isolation	Application; injection region; coordinates from bregma	Speed of application	Tau pathology	Propagation	References
C57BL/6 (endogenous tau); 3 m	11 m PI	Immunoprecipitated AD-tau oligomers (0.6 µg/site; 2 µl)	NA	Bilateral; Hippocampus *(A/P* = − *2.06 *mm, L = ± *1.75 mm, D/V* =− *2.5 mm)*	0.2 µl/min	Inclusion; Th-S positive structures	Hippocampus, frontal cortex, corpus callosum & hypothalamus	[96]
		Immunoprecipitated AD-PHF (0.6 µg/site; 2 µl)				Inclusions	No propagation	
ALZ17 (2N4R; WT; C57BL/6); 3 m	6 m, 12 m, & 15 m PI	Brain homogenate from AD (NA; 2.5 µl)	Temporal cortex	Unilateral; Hippocampus *(A/P* = *-2.5 mm, L* = + *2 mm, D/V* = *1.8 mm);* Overlying primary visual cortex; *(A/P* = *-2.5 mm, L* = + *2 mm, D/V* = *0.8 mm)*	1.25 µl/min	NFT- & NT-like; argyrophilic inclusions	Fimbria, optic tract, medial lemniscus, dorsal thalamus, cerebral peduncle amygdala, thalamus, internal capsule, entorhinal cortex, & fornix	[29]
		Brain homogenate from TD (NA; 2.5 µl)	Hippocampus			NFT- & NT-like; argyrophilic inclusions		
		Brain homogenate from AGD (NA; 2.5 µl)	Amygdala			NFT-like; argyrophilic grains		
		Brain homogenate from CBD (NA; 2.5 µl)	Globus pallidus			NFT-, NT-like, & astrocytic plaques; argyrophilic inclusions		
		Brain homogenate from PSP (NA; 2.5 µl)	Putamen			NFT-, NT-like; argyrophilic inclusion; tufted astrocytes		
		Brain homogenate from PiD (NA; 2.5 µl)	Frontal cortex			NFT-, NT-like; argyrophilic inclusions	No propagation	
PS19 (1N4R; P301S; (C57BL/6 × C3H)F1); 2–5 m	1 m, 3 m, & 6 m PI	Modified sucrose gradient enriched CBD-tau (0.05 µg; 2.5 µl/site)	Cortical grey matter	Unilateral; Hippocampus *(A/P* = *-2.5 mm, L* = + *2 mm, D/V* = *-2 mm)* Overlaying primary visual cortex *(A/P* = *-2.5 mm, L* = + *2 mm, D/V* = *-0.8 mm)*	NA	NT-like; oligodendroglial inclusions	CA2, CA3, dentate gyrus, fimbria, subiculum, thalamus, hypothalamus, & mammillary nuclei; no pathology in the overlying cortex	[19]
		Modified sucrose gradient enriched AD-tau (10.5 µg; 2.5 µl/site)				NT-like; inclusions	CA3, lateral septal nuclei, subiculum, white matter tracts with involvement of the fimbria, entorhinal cortex, locus coeruleus, raphe nuclei, supramammillary nuclei, neocortex, & contralateral hemisphere (CA3, entorhinal cortex)	
		Modified sucrose gradient enriched DSAD-tau (12.5 µg; 2.5/ µl)						
C57BL/6 (endogenous tau); 2–3 m	2 d,7 d, 1 m, 3 m, 6 m & 9 m PI	Sarkosyl insoluble AD-tau (4 μg/site; 2.5 μl/site) Sarkosyl insoluble AD-tau (1 μg/site; 2.5 μl)	Frontal cortical gray matter	Unilateral; Hippocampus *(A/P* = *-2.5 mm, L* = + *2 mm, D/V* = *-2.4 mm)* Overlying primary visual cortex *(A/P* = *-2.5 mm, L* = + *2 mm, D/V* = *-1.4 mm)*	NA	NFT-like; Th-S positive structures	Raphe nucleus, the mamillary area, locus coeruleus, fimbria, corpus callosum & both ipsilateral & contralateral dentate gyrus	[67]
C57BL/6 (endogenous tau); 15–19 m	1 m, 3 m, & 6 m	Sarkosyl insoluble AD-tau (1 μg; 2.5 μl)				NP-like; Th-S positive structures	Entorhinal cortex, locus coeruleus, corpus callosum, raphe nucleus, mammillary area & fimbria	
C57BL/6 (endogenous tau); 2–3 m	1 m, 3 m, 6 m, & 9 m PI	Sarkosyl insoluble AD-tau (2.25 μg/site; 2.5 μl/site)	Frontal cortical gray matter	Unilateral; Hippocampus *(A/P* = *-2.5 mm, L* = + *2 mm, D/V* =−*2.4 mm)* Overlying primary visual cortex *(A/P* = *-2.5 mm, L* = + *2 mm, D/V* =−*1.4 mm)*	NA	NFT-like	CA3, dentate gyrus, retrosplenial area, supramammillary nucleus, auditory cortex, & entorhinal cortex	[116]
		Sarkosyl insoluble CBD-tau (1.4 μg/site; 2.5 μl/site)	Frontal cortical gray & white matter			NFT-like; coiled bodies-like; astrocytic plaque-like	CA3, dentate gyrus, ventral hippocampus, fimbria, entorhinal cortex, corpus callosum, mammillary area, dorsal raphe, & olfactory bulb	
		Sarkosyl insoluble PSP-tau (2.5 μg; 4 μl)	Frontal cortical gray matter	Unilateral; Thalamus (Dorsal lateral geniculate nucleus) *(A/P* = *-2.5 mm, L* = + *2 mm, D/V* = *-3.4 mm)*		NFT-like; astrocytic plaque-like; tufted-astrocyte-like		
THY-Tau22 (1N4R; G272V & P301S; C57BL/6); 3 m	3 m PI	HMW sarkosyl Insoluble AD-tau (1 μg; 2 μl)	Frontal cortex	Unilateral; Hippocampus *(A/P* = *-2.1 mm, L* = + *1.5 mm, D/V* = *-2.0 mm)*	0.2 µl/min	Inclusions; argyrophilic grains	Ipsilateral CA1, CA3, dentate gyrus, fimbria & corpus callosum	[11]
C57BL/6 (endogenous tau); 3 m	3 m & 6 m PI					NT-like; coiled bodies-like; argyrophilic grains-like inclusions	CA1, CA2, the alveus, fimbria, & corpus callosum	
hTau (6 tau isoforms with endogenous tau-KO; C57BL/6); 3 m	6 m, 9 m, & 11 m PI	Sucrose gradient enriched AD p-tau oligomers (0.12 μg; 2.5 μl)	Cerebral cortex	Bilateral; Hippocampus *(A/P* = *-2.5 mm; L* = ± *2.0 mm; D/V* = *-1.8 mm)*	1.25 µl/min	NFT-like; NT-like	CA2, CA3, dentate gyrus, entorhinal cortex, subiculum, amygdala, corpus callosum, neocortex & septal nuclei	[74]
T40PL-GFP (2N4R; GFP-tagged P301L-tau; B6C3/F1); 2–3 m	3 m PI	Sarkosyl insoluble AD-tau (2 μg; 2.5 μl)	NA	Unilateral; *(A/P* = *-2.5 mm, L* = + *2 mm; D/V* =−*2.4 mm)*	NA	Inclusions	CA3, dentate gyrus, subiculum, retrosplenial granular cortex, entorhinal cortex, pons, & contralateral/ipsilateral hemisphere	[58]
C57BL/6 (endogenous tau); 2–3 m	3 m, 6 m & 9 m PI	Sarkosyl insoluble AD tau (2 µg/site; 5 µl)	Middle frontal gyrus	Unilateral; Hippocampus *(A/P* = *− 2.5 mm, L* = + *2 mm, D/V* = *− 2.4 mm*) Overlaying primary visual cortex *(A/P* = *− 2.5 mm, L* = + *2 mm, D/V* = *− 1.4 mm)*	0.4 µl/min	NFT-like	CA3, dentate gyrus, entorhinal cortex, retrosplenial area, supramammillary nucleus & auditory cortex	[15]
		Sarkosyl insoluble AD tau + Synthetic α-syn mpffs (5 µg/site; 2.5 µl)						
α-synKO (endogenous tau & α-syn -/-; C57BL/6); 2–3 m	3 m, 6 m & 9 m PI	Sarkosyl insoluble AD tau (2 µg/site; 5 µl)	Middle frontal gyrus	Unilateral; Hippocampus *(A/P* = *− 2.5 mm, L* = + *2 mm, D/V* = *− 2.4 mm*) Overlaying primary visual cortex *(A/P* = *− 2.5 mm, L* = + *2 mm, D/V* = *− 1.4 mm)*	0.4 µl/min	NFT-like	CA3, dentate gyrus, entorhinal cortex, retrosplenial cortex, supramammillary nucleus & auditory cortex	
C57BL/6 (endogenous tau); 10 m	6 m PI	Sarkosyl insoluble AD- tau (NA; 1.5 µl)	Hippocampus	Unilateral; Hippocampus *(A/P* = *-1.9 mm, L* = ± *1.4 mm, D/V* = *-1.5 mm)*	0.05 µl/min	NT-like; oligodendroglial inclusions	CA1, fimbria, septal nuclei, & periventricular hypothalamus	[45]
	6 m PI	Sarkosyl insoluble AD- tau (NA; 1.2 µl)	Hippocampus	Unilateral; Corpus callosum, *(A/P* = *-1.9 mm, L* = ± *1.4 mm, D/V* = *-1.0 mm)*	0.1 µl/min	NT-like; oligodendroglial inclusions	No propagation	
C57BL/6 (endogenous tau); 7 m & 10 m	4 m & 6 m PI	Sarkosyl soluble AD- tau (NA; 1.2 µl)	Hippocampus			No pathology	No propagation	
C57BL/6 (endogenous tau); m, & 10–12 m	4 m & 6–7 m PI	Sarkosyl insoluble GGT tau (NA; 1.2 µl)	Prefrontal cortex area 8			NT-like; oligodendroglial inclusions	Ipsilateral & contralateral corpus callosum	
C57BL/6 (endogenous tau); 12 m	6–7 m PI	Sarkosyl insoluble PART tau (NA; 1.2 µl)	Hippocampus			NT-like; oligodendroglial inclusions	Ipsilateral & contralateral corpus callosum	
		Sarkosyl insoluble ARTAG tau (NA; 1.2 µl)	Temporal white matter			NT-like; astroglial & oligodendroglial inclusions	Ipsilateral & contralateral corpus callosum	
C57BL/6 (endogenous tau); 10–12 m	6–7 m PI	Sarkosyl insoluble PSP tau (NA; 1.2 µl)	Striatum	Unilateral; Corpus callosum, *(A/P* = *-1.9 mm, L* = ± *1.4 mm, D/V* = *-1.0 mm*	0.1 µl/min	NT-like, & oligodendroglial inclusions	Ipsilateral & contralateral corpus callosum	
	6 m PI	Sarkosyl insoluble PiD tau (NA; 1.2 µl)	Hippocampus			NT-like, & oligodendroglial inclusions	Ipsilateral & contralateral corpus callosum	
		Sarkosyl insoluble fFTLD-P301L tau (NA; 1.2 µl)	Hippocampus			NT-like, & oligodendroglial inclusions	Ipsilateral & contralateral corpus callosum	
C57BL/6 (endogenous tau); 12 m	6–7 m PI	Sarkosyl insoluble GGT (P301T) tau (NA; 1.5 µl)	Frontal cortex	Unilateral; Hippocampus *(A/P* = *-1.9 mm, L* = *-/* + *1.4 mm, D/V* = *-1.5 mm*)	0.05 µl/min	NT-like; granular inclusions; coiled body-like	CA1, DG, fimbria, & corpus callosum	[49]
C57BL/6 (endogenous tau); 7 m	7 m PI	Sarkosyl insoluble GGT (P301T) tau (NA; 1.5 µl)	Subcortical white matter	Unilateral; Hippocampus *(A/P* = *-1.9 mm, L* = *-/* + *1.4 mm, D/V* = *-1.5 mm*)	0.05 µl/min	NT-like; granular inclusions; coiled body-like	CA1, DG, fimbria, & corpus callosum	
C57BL/6 (endogenous tau); 7 m & 12 m	4 m & 6–7 m PI	Sarkosyl insoluble GGT (P301T) tau (NA; 1.2 µl)	Frontal cortex	Unilateral; Corpus callosum *(A/P* = *-1.9 mm, L* = *-/* + *1.4 mm, DV* = *-1.0 mm*)	0.1 µl/min	NT-like; granular inclusions; coiled body-like	Ipsilateral middle & contralateral corpus callosum	
C57BL/6 (endogenous tau); 10 m	5 m PI	Sarkosyl insoluble GGT (P301T) tau (NA; 1.5 µl)	Frontal cortex	Unilateral; Caudate putamen *(A/P* = *0.14 mm, L* = *− /* + *2, D/V* = *− 2.5 mm)*	0.05 µl/min	NT-like; granular inclusions; coiled body-like	Restricted	
C57BL/6 (endogenous tau); 7 m	4 m PI	Sarkosyl soluble GGT (P301T) tau (NA; 1.5 µl)	Frontal cortex	Unilateral; Hippocampus CA2 *(A/P* = *-1.9 mm, L* = *-/* + *1.4 mm, D/V* = *-1.5 mm*)	0.05 µl/min	No pathology	Not applicable	
C57BL/6 (endogenous tau); 3–4 m	6–7 m	Sarkosyl insoluble sGGT/fGGT (K317M) tau (NA; 1.5 µl)	Frontal cortex	Unilateral; Hippocampus *(A/P* = *-1.9 mm, L* = *-/* + *1.4 mm, D/V* = *-1.5 mm*)	0.05 µl/min	NT-like; granular inclusions; coiled body-like	CA1, DG, fimbria, ipsilateral & contralateral corpus callosum	
C57BL/6 (endogenous tau); 3–4 m	6–7 m	Sarkosyl insoluble sGGT/fGGT (K317M) tau (NA; 1.5 µl)	Frontal cortex	Unilateral; Corpus callosum *(A/P* = *-1.9 mm, L* = *-/* + *1.4 mm, DV* = *-1.0 mm*)	0.1 µl/min	NT-like; granular inclusions; coiled body-like	CA1, DG, fimbria, ipsilateral & contralateral corpus callosum	
C57BL/6 (endogenous tau); 3 m & 7 m	3 m PI	Sarkosyl insoluble AGD-tau with no NFTs (NA; 1.5 µl)	Hippocampus	Unilateral; Hippocampus (*A/P* = *-1.9 mm, L* = *-1.4 mm, DV* = *-1.5 mm)*	0.05 µl/min	NT-like; granular inclusions; oligodendroglial inclusions	CA1, fimbria, & ipsilateral corpus callosum	[48]
C57BL/6 (endogenous tau); 3 m & 12 m	7 m PI					NT-like; granular inclusions; coiled body-like	CA1, fimbria, ipsilateral & contralateral corpus callosum	
C57BL/6 (endogenous tau); 7 m	3 m PI	Sarkosyl insoluble PART-tau (NA; 1.5 µl)				NT-like; granular inclusions; coiled body-like	Ipsilateral hippocampus, fimbria, & ipsilateral corpus callosum	
C57BL/6 (endogenous tau); 3 m & 12 m	7 m PI					NT-like; granular inclusions; coiled body-like	Ipsilateral hippocampus ipsilateral periventricular hypothalamus, septal nuclei, fimbria, ipsilateral, middle & contralateral corpus callosum	
C57BL/6 (endogenous tau); 7 m	3 m PI	Sarkosyl-soluble PART-tau (NA; 1.5 µl)				No pathology	Not applicable	
Tg601 (2N4R; WT-tau; C57BL/6); 2–3 m	17–19 m PI	Sarkosyl insoluble AD- tau (2 µg; 2.5 µl)	Frontal cortex	Unilateral; Hippocampus *(A/P* = *-2.5 mm, L* = ± *2.0 mm, D/V* = *-2.0 mm)*	0.25 µl/min	NFT-like; NT-like	Stratum lacunosum-moleculare of CA2, dentate gyrus, fimbria, CA1 pyramidal cell layer, external capsule, & dorsal raphe nucleus	[69]
Tg30tau (1N4R, P301S and G272V; C57BL/6); 1 m	5 w PI	Brain homogenate from AD (11 µg; 2 µl)	Frontal cortex	Unilateral; Hippocampus *(A/P* = *-1.8 mm, L* = *-1.72 mm, D/V* = *-1.8 mm)*	0.2 µl/min	NFT-like	Ipsilateral & contralateral regions of hippocampus	[4]
		Sarkosyl insoluble AD- tau (1 or 2 µg; 2 µl)				NFT-like	Ipsilateral & contralateral regions of hippocampus	
hTau (6 tau Isoforms with endogenous tau KO; C57BL/6); 2–3 m	3 m & 6 m PI	Sarkosyl insoluble FTDP-17 tau with P301L mutation (0.97 ug; 5-6 µl)	Frontal cortex	Unilateral; Hippocampus *(A/P* = *–2.5 mm, L* = + *2 mm, D/V* = *2.4 mm)*	NA	NFT-like; oligodendroglial & astrocytic inclusions	Ipsilateral & contralateral hippocampus, fimbria, corpus callosum, periaqueductal gray	[161]
		Sarkosyl insoluble FTLD-17 tau with E10 + 16 mutation (1 ug; 5-6 µl)				NFT-like, oligodendroglial & astrocytic inclusions	Ipsilateral & contralateral hippocampus, fimbria, corpus callosum, thalamus, hypothalamus, retrosplenial cortex, somatosensory cortex, auditory cortex, & supramammillary nucleus	
		Sarkosyl insoluble FTLD-17 tau with L266V mutation (0.6 ug; 5–6 µl)				NFT-like, oligodendroglial & astrocytic inclusions	Ipsilateral & contralateral hippocampus, fimbria, corpus callosum, thalamus, retrosplenial cortex, somatosensory cortex, & periaqueductal gray	
C57BL/6 (endogenous tau);–5 days	1 d, 2 d, 3 d, 1 m, 3 m, & 6 m PI	Sarkosyl insoluble AD- tau (3.84 µg; 1.2 µl)	Frontal cortex	Manually in thalamus	0.05 µl/min	NT-like, granular inclusions early on; at 3 m inclusions; at the age of 6, no pathology observed	No propagation	[47]
C57BL/6 (endogenous tau); 3 m	0 h, 1 d, 2 d, 3 d, 7d, 1 m, 3 m, & 6 m PI	Sarkosyl insoluble AD- tau (5.25 µg; 1.5 µl)		Unilateral; Ventral thalamus *(A/P* = *1.3 mm, L* = *− 1.2/ − 1.4 mm, DV* = *− 3/ − 3.5 mm)*		Granules inclusions, NT-like, & p-tau inclusions	Habenula, caudate/putamen, internal capsule, & fimbria	
	3 m PI	Sarkosyl soluble AD- tau (5.25 µg; 1.5 µl)				No pathology	No propagation	
C57BL/6 (endogenous tau); 1–5 d reinoculated at 3 m	3 m & 6 m PI	Sarkosyl insoluble AD- tau (3.84 µg; 1.2 µl); reinoculated sarkosyl insoluble AD- tau (5.25 µg; 1.5 µl)		Manually in thalamus; followed by reinoculation Unilateral; Ventral thalamus *(A/P* = *1.3 mm, L* = *− 1.2/ − 1.4 mm, DV* = *− 3/ − 3.5 mm)*		Granular inclusions	Caudate/putamen & corpus callosum	
C57BL/6 (endogenous tau); 6 m	3 m PI	Sarkosyl insoluble AD- tau (0.01 µg; 1.5 µl)	Hippocampus	Unilateral; Hippocampus *(A/P* = *− 1.9 mm interaural, L* = *− 1.4 mm, DV* = *− 1.8 mm)*	0.05 µl/min	NFT-like & granular inclusions	CA2, dentate gyrus, stratum radiatum, stratum oriens, corpus callosum, fimbria, entorhinal cortex, & cerebral cortex	[7]
6hTau (6 tau isoforms with endogenous tau-KO; C57BL/6); 6 m	3 m PI					NFT-, pre-tangles, & granular inclusions	CA2, dentate gyrus, stratum radiatum, stratum oriens, hilius, corpus callosum, fimbria, entorhinal cortex & cerebral cortex	
mtWT (endogenous tau KO; C57BL/6); 3 m	3 m PI6 m PI					No pathology	Not observed	
C57BL/6 (endogenous tau); 3 m	6 m PI	Sarkosyl insoluble AD- tau (Brain 1 = 0.5 μgBrain 1, 2, 3, & 3(concentrated) = 1- 4 μg; 2.5 µl/site)	Isocortex	Unilateral; Hippocampus *(A/P* = *− 2.5 mm, L* = *2 mm, D/V − 2.4 mm)* Overlaying primary visual cortex *(A/P* = *− 2.5 mm, L* = *2 mm, D/V − 1.4 mm)*	0.25 µl /min	NFT-like, NT-like	CA2, CA3, DG, fimbria, corpus callosum, retrosplenial area, parietal cortex, somatosensory cortex, entorhinal cortex & similar pattern in contralateral hemisphere	[120]
htau-App^NL−F/NL−F^ (6 tau isoforms with endogenous tau KO and mutant APP; C57BL/6) 3 m		Sarkosyl insoluble AD- tau (Brain 1 + 2 = 0.5 μg/site;.5 µl/site)						
Ptk2b KO; (endogenous tau with Ptk2b -/-; C57BL/6) 3 m		Sarkosyl insoluble AD- tau (Brain 1 + 2 = 0.5 μg/site; 2.5 µl/site)						
Tmem106b KO (endogenous tau with Tmem106b -/-; C57BL/6) 19 m		Sarkosyl insoluble AD- tau (Brain 1 + 2 = 0.5 μg/site; 2.5 µl/site)						
Grn KO; (endogenous tau with Grn -/-; C57BL/6) 3 m		Sarkosyl insoluble AD- tau (Brain 1 + 2 = 0.5 μg/site; 2.5 µl/site)						
SHR72 (2N4R, truncated tau aa151–391; SHR) 2 m	4 m PI	Sarkosyl insoluble AD- tau (600 ng; NA)	Parietal cortex	Bilaterally; Hippocampus *(A/P* = *− 3.6 mm, L* = ± *2.0 mm, D/V* = *− 2.3 mm)*	1.25 μl/min	NFT-like; argyrophilic inclusions	Rostral and caudal to site of injection in CA1	[140]
	2 m PI	Sarkosyl insoluble AD- tau from 3 independent brains (600 ng; NA)		Unilaterally; Hippocampus *(A/P* = *− 3.6 mm, L* = ± *2.0 mm, D/V* = *− 2.3 mm)*			Contralateral hippocampus	
	4 m PI	Solubilised sarkosyl insoluble AD- tau (600 ng; NA)		Bilaterally; Hippocampus *(A/P* = *− 3.6 mm, L* = ± *2.0 mm, D/V* = *− 2.3 mm)*		No pathology	Not applicable	
	4 m PI	Sarkosyl insoluble AD- tau (400/600 ng; NA)		Unilaterally; Hippocampus *(A/P* = *− 3.6 mm, L* = ± *2.0 mm, D/V* = *− 2.3 mm)*		NFT-like; argyrophilic tau inclusions	Contralateral hippocampus	
P301ST43 (1N4R; P301S; C57BL/6) 3 m	4 m PI	AD CSF tau (1 ng; 5 μl)	CSF	Unilateral; Hippocampus *(A/P* = *− 2.5 mm, L* = *− 2.0 mm, D/V* = *− 1.8 mm)*	1.25 μl/min	NFT-like & dot-like inclusions	Ipsilateral CA2, CA3, dentate gyrus, & contralateral hippocampus	[138]
6hTau (6 tau isoforms with endogenous tau-KO; C57BL/6) 3–5 m	1, 3 & 6 m PI	Sarkosyl insoluble AD-tau (1 µg/site; NA)	Frontal cortex	Unilateral; Hippocampus *(A/P* = *− 2.5 mm, L* = + *2 mm, D/V* = *− 2.4 mm)* Overlaying primary visual cortex *(A/P* = *− 2.5 mm, L* = + *2 mm, D/V* = *− 1.4 mm)*	NA	NT-like, NFT-like inclusions; argyrophilic inclusions	Ipsi- & contralateral CA1, CA2, dentate gyrus, subiculum, motor cortex, entorhinal cortex, visual cortex, thalamus, hypothalamus, corpus collosum	[71]
		Sarkosyl insoluble PiD-tau (1 µg/site; NA)				NFT-like; oligodendroglial inclusions; argyrophilic inclusions	Ipsi- & contralateral CA1, CA2, dentate gyrus, fimbria, thalamus, entorhinal cortex, visual cortex	
		Sarkosyl insoluble CBD-tau (1 µg/site; NA)				NFT-like; NT-like; argyrophilic inclusions; astrocytic & oligodendroglial inclusions	Ipsi- & contralateral CA1, CA2, dentate gyrus, fimbria, subiculum, thalamus, hypothalamus, corpus collosum, entorhinal cortex, visual cortex	
		Sarkosyl insoluble PSP-tau (1 µg/site; NA)				NFT-like; argyrophilic inclusions; astrocytic & oligodendroglial inclusions	Ipsi- & contralateral CA1, CA2, dentate gyrus, fimbria, corpus collosum, thalamus, hypothalamus, entorhinal cortex, visual cortex	
T44mTauKO (0N3R human tau; endogenous tau KO); 3–5 m	1, 3 & 6 m PI	Sarkosyl insoluble AD-tau (1 µg/site; NA)	Frontal cortex	Unilateral; Hippocampus *(A/P* = *− 2.5 mm, L* = + *2 mm, D/V* = *− 2.4 mm)* Overlaying primary visual cortex *(A/P* = *− 2.5 mm, L* = + *2 mm, D/V* = *− 1.4 mm)*	NA	No pathology	NA	
		Sarkosyl insoluble PiD-tau (1 µg/site; NA)				NFT-like; oligodendroglial inclusions	NA	
		Sarkosyl insoluble CBD-tau (1 µg/site; NA)				No pathology	NA	
		Sarkosyl insoluble PSP-tau (1 µg/site; NA)				No pathology	NA	
TauKDn^cre;fl/fl^ (Neuron specific tau KO; C57BL/6); 2–3 m	1,3,6 & 9 m PI	Sarkosyl insoluble PSP-tau (1 µg/site; NA)	Frontal cortex	Unilateral; Hippocampus *(A/P* = *− 2.5 mm; L* = + *2.0 mm; D/V* = *-2.4 mm)* Overlaying primary visual cortex *(A/P* = *− 2.5 mm; L* = + *2.0 mm; D/V* = *-1.4 mm)*	NA	No pathology	Not applicable	[115]
		Sarkosyl insoluble CBD-tau (1 µg/site; NA)				Astrocytic plaque-like; coiled body-like	Ipsi- & contralateral CA2, CA3, dentate gyrus, fimbriae, corpus callosum, visual cortex	
		Sarkosyl insoluble PSP-tau (1 µg/site; NA)				Tufted astrocytes; coiled body-like		
Tau.P301L (2N4R; P301L-tau; C57BL/6) 3 m	7 d and 28 d PI	Sarkosyl insoluble AD-tau (NA; 2 µl)	Frontal cortex	Unilateral; Hippocampus *(A/P* = *− 1.83 mm; L* = + *1.29 mm; D/V* = + *1.7 mm)*	0.25 µl/min	NFT-like	Hippocampal formation, cortex, corpus callosum, alveus of ipsi- & contralateral hemisphere	[38]
hTau (6 tau isoforms with endogenous tau-KO; C57BL/6); 3 m	1 & 12 m PI	Brain homogenate from CBD; (8 μg; 2 μl)	Frontal cortex	Unilateral; Right Striatum *(A/P* = *0.8 mm posterior, L* = *1.95 mm, D/V* = *3.0 mm)*	0.2 μl/30 s	NT-like; astrocytic plaque-like; coiled body-like	Ipsi- & contralateral entorhinal striatum, contralateral striatum, corpus collosum	[171]
LRRK2G2019S mice (Endogenous tau; LRRK2 KI; C57BL/6); 3–4 m	1, 3, 6, & 9 m PI	Sarkosyl insoluble AD-tau; (1 μg/site; 2.5 μl/site)	Frontal/temporal cortex	Unilateral; Hippocampus *(A/P* = *− 2.5 mm, L* = + *2.0 mm, D/V* = *2.4*) Overlying cortex *(A/P* = *-2.5 mm, L* = + *2.0 mm, D/V* = *1.4 mm)*	0.4 μl/min	NFT-like; NT-like	CA2, CA3, entorhinal cortex, dentate gyrus, ventral tegmental area, ipsilateral supramammillary nucleus, ipsilateral perirhinal area, medial septal nucleus, parasubiculum, presubiculum, pontine reticular nucleus, & ipsilateral accessory olfactory bulb	[32]
hTau (6 tau isoforms with endogenous tau KO; C57BL/6); 9–11 m	2.5 m PI	Sarkosyl insoluble AD-tau; (0.55 µg; 2.0 µl)	Isocortex	Unilateral; Hippocampus *(A/P* = *− 2.5 mm, L* = + *2.0 mm, D/V* = *− 1.67 mm)*	1.25 µl/min	Inclusions	Ipsi- & contralateral regions of hippocampus	[109]
PS19 (1N4R; P301S; C57BL/6) 3 m	3 m PI	Sarkosyl insoluble AD-tau; (1 µg; 2.0 µl)	Frontal cortex	Bilateral; Hippocampus *(A/P* = *− 2.4 mm, L* = + *1.5 mm, D/V* = *− 1.6 mm)*	0.2 µl/min	NFT-like; NT-like	CA2, CA3, overlaying isocortex	[106]
		HMW tau 2.33 µl/site				NFT-like; NT-like	CA2, CA3, overlaying isocortex, peri-/entorhinal	

*NA* Not available; *PI* Post inoculation; *PHF* Paired helical filaments; *AD* Alzheimer’s disease; *sAD* Sporadic AD; *TD* Tangle-only dementia, *PiD* Pick disease, *AGD* Argyrophilic grain disease, *PSP* Progressive supranuclear palsy, *CBD* Corticobasal degeneration; *sGGT* Sporadic globular glial tauopathy; *fGGT* Familial globular glial tauopathy; *ThS* Thioflavin S; *NFT* Neurofibrillary tangles; *NP* Neuropil; *NT* Neuritic plaques; *HMW* High molecular weight (> 242 kDa); *mpffs* Mouse preformed fibrils; *KI* Knock in; *KO* Knock out; *A/P* Anterior/posterior; *L* Lateral; *D/V* Dorsal/ventral

**Table 2:** Summary of studies involving rodent brain-derived tau inoculation

Experimental animal; age at inoculation	Time of termination	Rodent derived brain inoculum (pathological tau);(mass; volume)	Application; injection region (coordinates from bregma)	Speed of application	Pathology	Propagation	References
ALZ17 (2N4R; WT; C57BL/6); 3 m	6 m PI	Brain homogenate from P301ST43 (1N4R, P301S-tau); (NA; 2.5 µl)	Unilateral; Hippocampus *(A/P* = *-2.5 mm, L* = + *2 mm, D/V* = *-1.8 mm)* Overlaying primary visual cortex; *(A/P* = *-2.5 mm, L* = + *2 mm, D/V* = *0.8 mm)*	1.25 µl/min	NFT-like; NT- like; coiled bodies; argyrophilic inclusions	Fimbria, thalamus, hippocampus, optical tract, medial lemniscus, zona incerta, cerebral peduncle, visual cortex, hypothalamus, superior colliculus, & substantia nigra	[30]
	12 m					Fimbria, thalamus, internal capsule, caudate putamen, somatosensory cortex, hippocampus, optical tract, medial lemniscus, zona incerta, cerebral peduncle, visual cortex, hypothalamus, amygdala, superior colliculus, substantia nigra, entorhinal cortex, & deep mesencephalic nucleus	
	15 m PI					Fimbria, thalamus, internal capsule, caudate putamen, somatosensory cortex, hypothalamus, amygdala, hippocampus, optical tract, medial lemniscus, zona incerta, cerebral peduncle, visual cortex, superior colliculus, substantia nigra, entorhinal cortex, deep mesencephalic nucleus, & pontine nuclei	
SHR72 (0N4R; truncated tau, aa151–391); 2 m	25 d & 3.5- 5 m PI	Sarkosyl insoluble from SHR24 (0N3R, truncated tau aa151–391); (0.1 µg; 2 µl)	Unilateral; Motor cortex 1; *(A/P* = + *3 mm, L* = + *2 mm, D/V* = − *0.75 mm)*	0.5 µl/min	NFT-like	Ipsilateral frontal cortical areas & striatum	[100]
SHR24 (0N3R, truncated tau, aa151–391); 2 m		Sarkosyl insoluble tau from SHR72 (0N4R tau, aa151–391); (0.04 µg; 2 µl)	Unilateral; Motor cortex 1; *(A/P* = + *3 mm, L* = + *2 mm, D/V* = − *0.75 mm)*		NFT-like	Ipsilateral frontal cortical areas, contralateral frontal cortex, & striatum	
P301ST43 (1N4R; P301S; C57BL/6) 2 m, 2.5 m, 3 m, 4 m, & 4.5 m	1 d, 14 d, 1 m, 2 m, & 2.5 m PI	Sarkosyl insoluble tau from P301ST43 (1N4R, P301S-tau); (0.04 ng; 2.5 µl)	Unilateral; Hippocampus *(A/P* = *-2.5 mm, L* = + *2 mm, D/V* = *-1.8 mm)* Overlying primary visual cortex; *(A/P* = − *2.5 mm, L* = + *2 mm, D/V* = − *0.8 mm)*	1.25 µl/min	NFT-like; NT-like; argyrophilic inclusions	CA3, dentate gyrus, contralateral hippocampus, subiculum & retrosplenial cortex, mammillary nucleus, supramammillary nucleus, thalamus, nucleus accumbens, & lateral septal nucleus	[1]
rTg4510 (2N4R; P301L; C57BL/6); 2–3 m	2 d & 21 d PI	Brain extract from rTg4510 (0N4R, P301L-tau, HMW); (0.25 µg; 2.5 µl)	Unilateral; Hippocampus *(A/P* =−*2.5 mm, L* = *2.0 mm, D/V* =−*1.8 mm)*	0.2 µl/min	NFT like inclusions	CA2, CA3, & dentate gyrus	[146]
		Sarkosyl insoluble tau from rTg4510 (0N4R, P301L-tau, LMW); (0.25 µg; 2.5 µl)			No pathology	No spreading	
P301ST43 (1N4R; P301S; C57BL/6); 2.5 m	2.5 m PI	Total brainstem lysates from P301ST43 (1N4R, P301S-tau); (NA; 2.5 μl)	Unilateral; Hippocampus *(A/P* = *-2.5 mm, L* = + *2.0 mm, D/V* = *1.8 mm)* Overlaying primary visual cortex; *(A/P* = − *2.5 mm, L* = + *2 mm, D/V* = − *0.8 mm)*	1.25 µl/min	NFT-like; NT-like	Ipsi- & contralateral CA2, dentate gyrus, subiculum, thalamus, mammillary nuclei & retrosplenial cortex	[80]
		40% sucrose gradient fraction from P301ST43 (1N4R, P301S-tau); (NA; 2.5 μl)			NFT-like; NT-like	Ipsi- & contralateral CA2, CA3, dentate gyrus, subiculum, thalamus, mammillary nuclei & retrosplenial cortex	
		10% fractions gradient fractions from P301ST43 (1N4R, P301S-tau); (NA; 2.5 μl)			NFT-like (low in numbers)	Ipsi- & contralateral CA2, CA3, dentate gyrus, & subiculum	

*NA* Not available; *PI *Post inoculation; *AD* Alzheimer’s disease; *TD* Tangle-only dementia, *PiD* Pick disease, *AGD* Argyrophilic grain disease, *PSP* Progressive supranuclear palsy, *CBD* Corticobasal degeneration; *mpffs* Mouse preformed fibrils; *KI* Knock in; *KO* Knock out; *A/P* Anterior/posterior; *L* Lateral; *D/V* Dorsal/ventral

**Table 3:** Summary of studies involving the inoculation of extracellular vesicles

Experimental animal; age at inoculation	Time of termination	EVs source (pathological tau);(mass; volume)	Application; injection region (coordinates from bregma)	Speed of application	Pathology	Propagation	References
C57BL/6 mice; 3-4 m	1 m & 2 m PI	Exosomes derived from neuronally-differentiated, human iPSCs contain human tau-RD-LM-YFP; 2.5 µg	Unilateral; Hippocampus *(A/P* = *-2.0 mm, L* = + *1.5 mm, D/V* = *-1.3 mm)*	0.5 µl/min	NFT-like	Ipsilateral thalamic nuclear regions (TH), piriform/entorhinal (Pir/EC) cortices and contralateral CA1	[163]
C57BL/6); 2 m	5w PI	Exosomes derived from neuronally-differentiated, human iPSCs from fAD patient harbouring an A246E mutation to PS1; 1.34 µg	Bilateral; Hippocampus *(A/P* = *-2.0 mm, L* = ± *1.75 mm, D/V* = *-1.75 mm)*	NA	Inclusions	No propagation	[12]
ALZ17 mice (2N4R; WT; C57BL/6); 3 m	6 m PI	4-6 m old WT mice; 2.5 µg	Bilateral; Hippocampus CA1 *(A/P* = *-2.5 mm, L* = ± *2 mm, D/V* = *-1.8 mm)*	0.25 µl/min	No pathology	NA	[13]
	6 m PI	4-6 m old rTg4510 mice; 2.5 µg			Oligomeric tau inclusions	Stratum radiatum, Schaffer collateral fibers from the CA3 to CA1 region	
C57BL/6 mice; 18-19 m	4.5 m PI	Human AD brain (frontal cortex) derived EVs; 0.0003 µg	Unilateral; Hippocampus DG *(A/P* = *-2.18 mm, L* = ± *1.13 mm, D/V* = *-1.9 mm)*	NA	Inclusions	Both ipsilateral and contralateral hippocampal region including the CA1, CA3, dentate granule cells, subgranular zone, and hilus	[131]
		Human prodromal AD brain (frontal cortex) derived EVs; 0.0003 µg					
THY-tau30; 1 m	1 m PI	AD BD-EVs 2 mL; 6 × 10^9^ vesicle	Bilateral; Hippocampus DG *(A/P* = − *2.5 mm, L* = ± *1 mm, D/V* = − *1.8 mm)*	0.2 mL/min	NFT-like	CA1	[99]
		PSP BD-EVs 2 mL; 6 × 10^9^ vesicle			No pathology	NA	
		PiD BD-EVs 2 mL; 6 × 10^9^ vesicle			No pathology	NA	

### Study characteristics

**Fig. 1:**
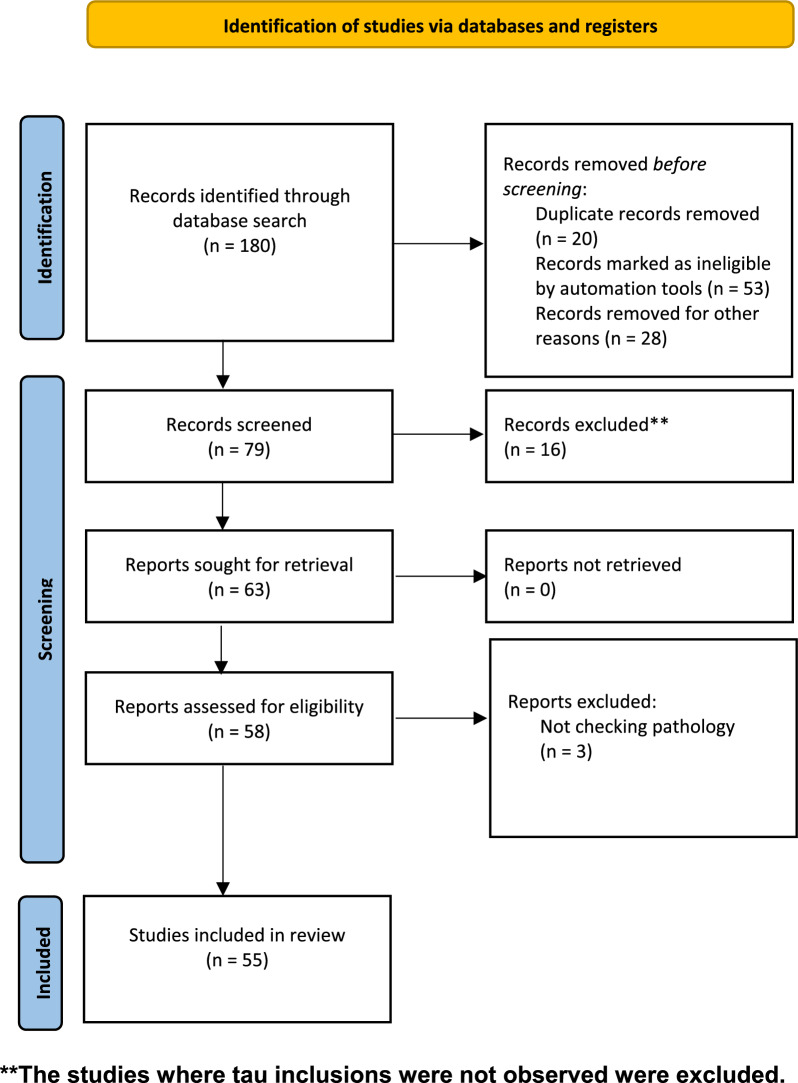
PRISMA flow diagram

Through a database search we identified 55 relevant studies that have utilized a wide range of tau propagation models for intracerebral inoculation to model tau propagation in vivo (Fig. 1).

## Results

### Deep insight into the tau inoculum

The biochemical and biophysical characterization of tau inoculum is the first vital step toward comprehending one half of the pathological process involved in its propagation. Based on its origin, the tau inoculum utilized in experimental tau propagation models can be categorized as follows:

1. Human brain-derived insoluble tau (free oligomeric or fibrillar tau)
2. Human brain-derived insoluble tau encapsulated in exosomes
3. Rodent brain-derived insoluble tau (free oligomeric or fibrillar tau)
4. Rodent brain-derived insoluble tau encapsulated in exosomes
5. Synthetic pre-formed fibrils.

**Fig. 2:**
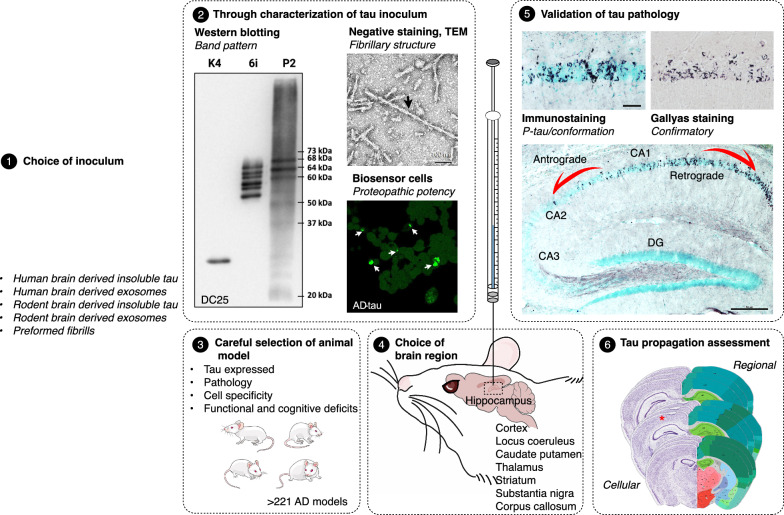
Inoculation-based tau propagation models. The graphical abstract illustrates the pivotal elements highlighted in this review. The central theme revolves around the transcellular propagation of aberrantly modified tau protein along the functional brain network. The depicted framework emphasizes the critical steps for establishing robust models, including the judicious selection and comprehensive characterization of tau inocula through functional, biochemical, and biophysical analyses (**1**, **2**). Key considerations involve the careful choice of animal models (**3**), optimal inoculation sites (**4**), the crucial validation of fibrillary pathology using confirmatory staining techniques (**5**) and downstream assessment (**6**). The proposed framework serves as a practical guide for researchers, offering a systematic approach to establish benchmark models for preclinical testing of potential disease-modifying drugs (DMTs)

Human brain-derived inocula were extracted from the isocortex, allocortex, amygdala, or subcortical nuclei of human Alzheimer’s disease [4, 7, 11, 15, 19, 29, 45, 47, 48, 58, 65, 67, 69, 71, 74, 96, 107, 115, 116, 120, 138, 140, 156, 161], Down syndrome (DS) indistinguishable from AD (DSAD) [19], argyrophilic grain disease (AGD) [29, 48], Pick’s disease (PiD) [29, 45, 71], tangle-only dementia (TD) [29], globular glial tauopathies (GGT) [45, 49], corticobasal degeneration (CBD) [19, 29, 71, 115, 116, 171], primary age related tauopathy (PART) [45], aging-related tau astrogliopathy (ARTAG) [45], frontotemporal dementia with parkinsonism linked to chromosome 17 (FTLD-17) [45, 161], and progressive supranuclear palsy (PSP) [29, 45, 71, 115, 116] brains (Fig. 2). The choice of region from which the inoculum was isolated was based on the anatomical distribution of tau inclusions, which varies greatly depending on the disease and its stage. In fact, tau from a single brain can exhibits variation in its proteopathic potency based on the region [82], or within the region whether it is derived from grey or white matter [165], moreover fractions show differences even within a single isolation [102]. Additionally, the tau inclusions have distinct morphological characteristics and cell-specificities [28, 114] and are composed of either 3R, 4R, or both isoforms with disease-specific filament folds [43, 44, 137, 147, 176].

Rodent brain-derived inocula were extracted from the cortices, brainstems, or spinal cords of transgenic mice (P301ST43 [1, 30, 80], rTg4510 [146]), or rats (SHR24 [100], SHR72 [100]), taking into account the regional distribution of tau inclusions. These inclusions are driven by the transgenic expression of either human mutant tau such as P301S [5], or P301L [128, 134] or by truncated tau (aa151-391) found in sporadic AD [51, 178] and are composed of both transgenic and if not knocked out, endogenous rodent tau. The 3R/4R isoform and the filament-fold are either reported to be NFT-like or remains to be determined at large.

From both human and rodent brains, extracellular vesicles, particularly exosomes, were derived from prodromal AD, mild cognitive impairment (MCI), AD, PSP or PiD [99, 131] or from the iPSCs derived from AD patients [12, 163], as well as from brain extracts from Tg rodent models of tauopathy [13]. Given that the neuronal cells are recognized for producing exosomes that transport cargo such as proteins, RNA-binding proteins (RBPs), and RNAs to neighbouring cells, exosomes were initially considered and subsequently confirmed to be vehicles facilitating the cell-to-cell transmission of oligomeric tau [16, 125]. The 3R/4R isoform composition of tau with characteristic filament-folds within these exosomes is specific to the disease or model.

Human/rodent brain-derived insoluble tau inoculums were prepared as either enriched detergent-insoluble protein aggregates or, in some cases, as crude protein extracts (Tables 1, 2, & 3). It is imperative to underscore that within the widely utilized sarkosyl-insoluble protein aggregate fraction, tau comprises a mere 10% of the total proteins [39]. This fraction encompasses additional constituents, including β-amyloid (Aβ), snRNP70 (U1-70 K), apolipoprotein E (ApoE) and complement component 4 (C4-A), among others [39, 64]. With the aim of obtaining a homogenous sample of aggregated tau, certain studies have also incorporated, additional fractionation steps, employing techniques such as immunoprecipitation (IP) or fast protein liquid chromatography (FPLC) [9]. These fractions also consisted of a diverse pool of fibrillary, filamentous, multimeric, and oligomeric units of pathological tau.

The propagation potential of oligomers, ranging from 3 to 100 molecules (< 30 nm long fibrils) [26, 80, 110, 160] along with larger insoluble tau aggregates [80] is ongoing. Although, it remains unclear which molecular entity among these is capable of propagation, tau aggregates were broken down into smaller fragments through differential sonication protocols before inoculation. This step is aligns with the prevailing notion that a soluble, high-molecular-weight (HMW) oligomeric form of tau may exhibit comparable or even heightened bioactivity in terms of propagation across neural networks [106, 146] (Tables 1, 2 and 3).

The comprehensive characterization of brain-derived tau inocula involved a multifaceted approach employing various techniques. Initial assessments utilized Western blot (WB) [15, 29, 45, 48, 69, 74] and enzyme-linked immunosorbent assay (ELISA) [1, 4, 15, 19, 116, 138, 166, 176]. Some studies opted for the bicinchoninic acid (BCA) assay [1, 4, 7, 45–48, 58, 67, 116], nanodrop spectrophotometry [120, 166] for total protein estimation. Further biophysical characterization of the tau inoculum employed circular dichroism (CD) spectroscopy [96], transmission electron microscopy (TEM) [11, 47, 116, 140, 164], atomic force microscopy (AFM) [95, 120, 146], fast protein liquid chromatography (FPLC) [95], immunoelectron microscopy (IEM) [30], and size exclusion chromatography (SEC) [42, 67, 99]. In the preparation of exosomes, the human/rodent brain homogenates were subjected to sequential differential centrifugation, ultracentrifugation, and ultrafiltration or size exclusion chromatography (SEC) to further purify and concentrate the extracellular vesicles. While the proteomic composition of these vesicles exhibits considerable variability, it has been reported that tau is enriched among the cargo they carry. Furthermore, in order to determine the size and abundance of the exosomes, nanoparticle tracking analysis (NTA) was performed [12, 131, 163], followed by characterization using silver gel staining, electron microscopy, proteomics, and WB/ELISA of the exosome lysate or AFM to validate the presence of tau oligomers.

WB analysis provided insights into the distinctive band patterns of the 3R and 4R tau isoforms, along with disease-specific phospho-tau signature. In the case of AD, the fraction typically exhibited characteristic band patterns with three bands at 68, 64, and 60 kDa, accompanied by a weak upper band of approximately 73 kDa. Contrarily, fractions related to ARTAG, GGT, PSP, and FTDP-17 exhibited two bands of 68 and 64 kDa, which are specific to 4R tau tauopathies [25]. PiD, in contrast, exhibited two bands at 64 and 60 kDa, distinctive of 3R tau tauopathies [62]. Additionally, several lower bands indicated the presence of a fragmented pool of tau spanning between 50 and 25 kDa [62].

The exact concentration of tau was determined through ELISA, while a rough estimation of total protein was made using BCA or nanodrop spectrophotometers. Notably, across the studies, different amounts were inoculated, and as expected, a “dose-dependent” effect on the resulting pathology load was observed [8, 107]. This suggests that the progression of tau pathology is influenced by the quantity of propagation-capable tau in the inoculum, with the understanding that this relationship may not necessarily follow a linear pattern [108]. To confirm the presence of disease-specific fibrillary cores, the monomers-to-oligomers ratio, and the size of fragmented fibrils, techniques such as AFM, IEM, TEM, SEC, and CD spectroscopy were employed. Finally, in vitro validation of the proteopathic potency of tau prior to inoculation involved the use of either Tau RD fluorescence resonance energy transfer (FRET)-based biosensor cells [72] or primary neurons [117].

Synthetic PFFs, have played a pivotal role in demonstrating that tau-aggregation and propagation can be induced by altered tau alone, independent of other proteins, underscoring the role of pathologically altered tau as the causative agent. These fibrils were meticulously characterized and were composed of recombinantly produced full-length (T40) [58, 67, 75, 76, 164] or truncated (K18, K19, dGAE) tau [4, 75, 123, 144, 155, 162, 173], either as wild type [76, 162, 164, 173] or with mutations (P301S [4, 58, 75, 123, 144, 155], P301L [75, 76, 103] or C291S[164]). They were assembled into fibrillary aggregates using polyanionic inducers such heparin [4, 58, 67, 75, 76, 103, 123, 133, 144, 155, 162, 164, 173]. Aggregation confirmation was obtained through Thioflavin T assay [4, 67, 75, 76, 103, 155], or TEM [4, 67, 76, 144, 155, 164, 173]. These PFFs have been validated in both cellular and in diverse in vivo models, inducing robust tau pathology and propagation of tau pathology, thereby offering a comprehensive window for analysis [123, 142, 143, 159]. However, recent cryo-EM studies indicates that the core of the in vitro induced fibrillary structure differs from that of human fibrils [175], potentially limiting their translational value to a certain extent. This disparity might be attributed to the specific fragment used or to the potential lack of some posttranslational modifications, as these have been predominantly produced in *E. coli* expression system. Nevertheless, *Baculovirus* or mammalian expression systems could be utilized to counteract the lack of crucial post-translational modification (PTMs) in recombinant tau, such as N-linked or O-glycosylation and widespread Ser/Thr phosphorylation in prokaryotic systems [63].

In summary, while it is essential to acknowledge that the diverse inocula have their unique value and significance in studying different aspects of AD and related tauopathies, among the aforementioned five types of inoculums, the disease-specific human brain-derived tau fibrillary assemblies in either free or encapsulated form exhibit a notably close resemblance to pathological characteristics and the propagation behaviour. To that end, single cycle in vitro amplification of human disease-specific bioactive tau (AD-, PSP- and CBD-lysate) is proposed as an alternative to minimize the reliance on human brain-derived tau and reduce variability induced by the utilization of inoculums from different brains. This would allow extending the scale of the experiments for future studies, and increase comparability [166]. Moving forward, a comprehensive biochemical and biophysical characterization of tau inocula utilized in experiments will enable us to identify the specific attributes such as tau isoforms, hyperphosphorylation patterns, truncations, disease-associated filament-fold changes, and aggregation states (monomers, oligomers, or larger fibrillar aggregates). These characteristics are crucial for establishing comparability and reproducibility across studies, contributing to a more comprehensive understanding of tau pathology.

### In vivo recapitulation of the tau pathology

In tau propagation studies, a recurrent challenge involves recapitulating *bona fide* pathological inclusions post-inoculation. Although caution should be taken to extrapolate findings based solely on the induction of tau phosphorylation, which does not necessarily indicate dysfunction of tau protein or its relevance for a pathological phenotype [89]. Therefore, it is imperative to develop a standardized protocol for recapitulating fibrillary-like inclusions akin to those observed in human tauopathies. In order to discern the most optimal methodology, we briefly summarize the techniques employed for validating tau inclusions, as well as the diverse types of inclusions observed in various regions with distinct tau inoculum in the studies conducted thus far.

In AD and other tauopathies, immunohistochemistry (IHC) and conventional histological staining techniques are the gold-standard methods for characterizing tau lesions. The primary antibody frequently used in IHC is AT8 (targeting p-tau pS202/pT205) [2, 20, 111]. Additional antibodies also include — RD3 (aa 209–224) [59, 88] and RD4 (aa 275–291) [59, 88] for different tau isoforms, PHF‐1(p-tau Ser396/Ser404) [20, 111] and CP13 (p-tau Ser202) [20] for phospho-tau, MC‐1(aa 312–322) for pathological conformation [20], TauC3 for detecting tau truncated at aspartic acid 421, and Tau‐66 (aa 155–244, 305–314) and Alz‐50 (aa 2–10 and 312–342) [20] for specific tau epitopes. Conventional histological staining methods utilize Gallyas silver stain [2, 3, 23] and Thioflavin S [23, 124], with Bielschowsky [2, 3] and Campbell‐Switzer [23] staining less commonly employed. These staining techniques are crucial for identifying the β-pleated structures that constitute the core of tau fibrils, thus validating the presence of mature pathological aggregates.

In the shortlisted studies, an array of anti-tau antibodies were utilized to characterize pathological inclusions. These antibodies encompassed phosphorylation-dependent variants, notably AT8 (p-tau Ser202/Thr205) [4, 7, 11, 15, 19, 29, 45, 58, 67, 69, 96, 120, 138, 140, 146], PHF 1 [7, 11], AT100 (aa p-tau Thr212 and Ser214) [4, 11, 29, 138], AT180 (p-tau Thr231)[7], T14 (tau and p-tau of A68 polypeptides) [19, 29], and 12E8 (p-tau Ser262 and/or Ser356) [29, 74]. Additionally, conformation-sensitive antibodies such as MC1 (aa 7–9, aa 312–322) [1, 7, 11, 19, 67], isoform-specific antibodies for 3 repeat tau (RD3) [7, 19] and 4 repeat tau (RD4) [7, 11, 19], along with mouse specific tau antibody T49 [19, 58], MT1 [29], and R2295 [15, 58]; and a human-specific pan-tau antibody, HT7 (aa 159 and 163) [15, 67, 96] were employed for comprehensive characterization. The confirmation of fibrillary pathology was conclusively established through Gallyas silver staining [11, 29, 138, 140], thioflavin S staining [19, 67, 69, 74, 120, 146], or a combination of both [1, 96].

In AD, neurofibrillary pathology manifests as pre-tangles, neurofibrillary tangles (NFTs), neuritic plaques (NPs), neuropil threads (NTs), and ghost tangles [2, 21]*.* To investigate the pathobiology in vivo, it is essential that propagation models mimic the aforementioned disease-associated neurofibrillary inclusions. The induction of tau pathology using AD-tau inoculum successfully recapitulated NFT-like tau inclusions and NTs in mouse models such as ALZ17 [29] and hTau [71, 74]. Similarly, our group previously reported the presence of robust NFT-like tau inclusions and NTs in transgenic rat models, SHR72 and WKY72 [107, 139, 140]. Some studies also noted the presence of NPs [120], globular deposits [7] in WT mice, oligodendroglial tau inclusions in both WT [47] and ALZ17 mice [29], as well as argyrophilic grains in WT [48], THY-T22 [11] and ALZ17 mice [29]. However, several other studies solely noted the presence of aberrantly hyperphosphorylated tau, which does not meet the criteria of neurofibrillary tau pathology.

In CBD, the pathological features encompass corticobasal bodies, astrocytic plaques, neuronal threads, oligodendrocytic coiled bodies, twisted tubules in oligodendroglial cells, and small NFTs [88, 135]. Upon inoculation of CBD-tau, hTau mice developed NFT-like pathology, astrocytic and oligodendroglial tau inclusions [71, 171]. ALZ17, on the other hand, developed NFT-like pathology and astrocytic plaques, but did not recapitulate oligodendroglial tau inclusions [29]. In both WT and PS19 mice, the pathology manifested as astrocytic plaques and oligodendroglial inclusions [29, 71, 116], while with neuronal tau knocked out only oligodendroglial tau inclusions were observed [115].

In PSP, the pathological features encompass the presence of NFTs and threads in subcortical nuclei, tufted shape astrocytes, oligodendroglial coiled bodies, and diffused cytoplasmic structures in neurons [88, 90]. Upon inoculation of PSP-tau in 6hTau, ALZ17, and WT mice, the observed pathology included NFTs, and astrocytic and oligodendroglial tau inclusions [29, 71, 116]. Additionally, mice with knocked out tau expression in neurons developed only oligodendroglial tau inclusions [115].

In PiD, the pathological features encompass neuronal Pick bodies, Pick cells, ramified astrocytes, and globular inclusions in oligodendrocytes [57, 87, 154]. Upon inoculation of PiD-tau, ALZ17 mice [29], hTau and T44mTauKO [71] developed NFTs and oligodendroglial argyrophilic inclusions. WT mice developed a lower pathological load compared to T44mTauKO and hTau mice [71]. The heightened load in T44mTKO can be attributed to two factors: (1) there is no transgenic expression of human 3R tau in ALZ17, and cross inoculation experiments have shown that 4R tau could not efficiently recruit 3R tau [67]; (2) the transgene expression level in T44mTauKO exceeds that of AL17 by several folds [71].

A significant number of studies are confounded by the use of single antibody, or no confirmatory staining. Assertions of inducing tau pathology are widespread, yet caution should be taken to conflate tau phosphorylation, often in the form of a diffuse signal in the cytoplasm, with neurofibrillary pathology. The nomenclature in this context should be clarified, designating diffuse phosphorylation as such, while the term “tau pathology” should be reserved for actual tau aggregates containing beta-sheets. It is noteworthy that the standard confirmatory staining methods, such as Gallyas and Thioflavin S, can serve as robust evidence to validate tau lesions. However, validation can be further enhanced by incorporating techniques such as transmission electron microscopy (TEM) and, ideally, cryo-electron microscopy (cryo-EM) to confirm the presence of filaments [156] and elucidate their structure at atomistic resolution [137].

Taking into consideration the existing evidence, it can be concluded that recapitulating tau pathology is feasible in inoculation-based tau propagation models using either transgenic or non-transgenic models. It is noteworthy that while transgenic models in studies recapitulating CBD, GGT, AGD, and PSP predominantly utilize neuron-specific promoters like Thy1 [5, 126, 149], Thy1.2 [122], murine prion promoter (Prnp) [77, 170], CaMKIIα [85], or human tau promoter [6], they have consistently reproduced tau inclusions in oligodendrocytes and astrocytes in tau propagation models, albeit in small quantities. This suggests that tau overexpression is crucial for robust pathology recapitulation. Employing a model exclusively expressing tau in glial cells could offer a more effective approach to mimic tauopathies with predominant glial tau pathology. The judicious selection of models and histopathological validation of tau inclusions in regions where propagation occurs holds utmost importance. While every model possesses unique strengths, in the context of recapitulating authentic tau pathology, optimal choices include ALZ17, htau, SHR72, and WKY72 for AD; hTau and ALZ17 for PSP; and ALZ17 and PS19 for CBD. It's noteworthy that, none of these models can be used to successfully recapitulate full blown Pick-like pathology.

### Selective regional vulnerability of tau propagation

In light of the data from human research showing that tau propagation follows connectivity patterns in the brain [53, 55], we sought to investigate to what extent the relationships between tau burden and functional connectivity observed in distinct human tauopathies are faithfully recapitulated in the inoculation tau propagation models.

The hippocampus emerged as the preferred site of tau inoculation across the majority of studies [1, 4, 10, 11, 15, 19, 29, 30, 32, 45, 48, 49, 58, 67, 69, 74, 75, 80, 96, 109, 116, 123, 144, 146, 157, 161, 162, 173]. This preference is rooted in the understanding that the neurofibrillary pathology in the AD brain initiates within the hippocampus, subsequently propagating sequentially to the limbic system and ultimately reaching the isocortex [21]. In a few studies, tau inoculation into the hippocampus was followed by inoculation into the overlying cortex [1, 15, 19, 29, 30, 32, 65, 67, 71, 80, 109, 115, 116, 120, 155]. While some studies have focused on isolated cortical injections [100, 164], others explored the propagation from other brain structures such as the locus coeruleus [76], caudate putamen [49], thalamus [47, 116], striatum [75, 171], substantia nigra [144], and corpus callosum [45, 49].

Upon injection in the hippocampus (CA1/CA2/CA3) or dentate gyrus (DG), fibrillary tau pathology propagated throughout the anatomically and functionally connected regions. The hippocampus projects to the mammillary bodies through afferent fibers in the fornix. The mammillary bodies innervate the anterior thalamic nucleus and tegmental nuclei, connecting other structures to the frontal cortex and brain stem, respectively (76). The CA1 and entorhinal cortex (EC) regions heavily innervate the subiculum, which is one of the major sources of efferent projections from the hippocampal formation. In line with this far-reaching network of hippocampal connections, the CA regions [1, 4, 7, 11, 15, 19, 30, 32, 45, 48, 49, 58, 67, 69, 71, 75, 80, 96, 109, 115, 116, 120, 123, 138, 140, 144, 146, 155, 161, 162, 173], DG [1, 7, 11, 15, 32, 49, 58, 67, 69, 71, 75, 80, 109, 115, 120, 123, 138, 142, 146, 173], the fimbria [4, 7, 11, 19, 29, 30, 32, 45, 48, 49, 67–69, 71, 115, 120], the entorhinal cortex (EC) [1, 7, 15, 19, 58, 67, 71, 75, 116, 120, 144, 173], and subiculum [1, 19, 32, 58, 71, 74, 80] were all reported positive for tau inclusions. Some studies also found associated proximal regions such as fornix [29], and mammillary area [1, 15, 19, 32, 67, 80, 116, 161] being affected.

Upon extended post-inoculation observation time (≥ 9 months), regions connected to the hippocampus across the whole brain were found affected, as evident from the presence of tau lesions in cortical regions [15, 71, 74, 96, 115, 116, 144], along with others such as amygdala [29, 30, 144], thalamic nuclei [19, 29, 30, 71, 96, 144], external capsule [69], internal capsule [29], septal nuclei [32, 74], hypothalamus [30, 96], optic tract [29], olfactory bulb [32, 116], and nuclei in the brain stem [19, 30, 67, 69, 116, 161].

In the studies with unilateral injections, the contralateral side was consistently positive for tau inclusions due to the apparent involvement of the corpus callosum (CC) [1, 4, 7, 11, 19, 38, 45, 48, 49, 58, 67, 71, 80, 96, 109, 115, 116, 120, 138, 140, 144, 155, 161, 162, 171, 173]. Whereas, when tau was inoculated in the cortex, pathology was observed at the site of injection, and the contralateral cortex [100]. Similarly, to show different regional vulnerability, upon tau inoculation in the thalamus or locus coeruleus, propagation was observed throughout the functionally connected regions [47, 76, 116]. On the contrary, no propagation was reported from the caudate putamen following inoculation there [49].

Notably, nuclei that are not directly connected to the hippocampal formation did not show evidence of tau propagation following inoculation into the hippocampus – whether this was due to time, or due to the inability of the inocula to induce pathology in a sequence of linked brain regions is unknown. The mode of propagation of pathological tau isolated from various tauopathies has demonstrated contradictory results. While some reported tau propagate in a similar manner regardless of the disease-specific inoculum [15, 29, 45, 48, 71], others showed different patterns of propagation [19, 161]. In a nutshell, both anterograde and retrograde propagation of tau in both transgenic and WT models are related more to the strength of the connectivity of neuronal networks between the inoculation region and another brain region, rather than to the proximity of the brain region to the injection site [127].

The hippocampal formation and associated areas assist with spatial and episodic memory consolidation and storage. Accordingly, some tau inoculation models described above exhibit behavioral deficits when compared to those in sham-treated animals [11, 15, 69], suggesting that these models are suitable for inclusion in behavioral assessments as an efficacy measure in preclinical efficacy studies (Tables 4 and 5).

**Table 4:** Summary of studies that involve the inoculation of synthetic in vitro synthesized PFFs

Experimental animal; age at inoculation	Time of termination	Pathological tau (mass; volume)	Application; injection region (coordinates from bregma)	Speed of application	Pathology	Propagation	References
C57BL/6; 2–3 m	3,6,9, 18, & 24 m PI	Heparin-treated 2N4R tau PFFs (9 µg; 5 µl)	Unilateral; Hippocampus *(A/P* = *-2.54 mm, L* = + *2 mm, D/V* = *-2.4 mm)* Overlaying primary visual cortex *(A/P* = *-2.54 mm, L* = + *2 mm, D/V* = *-1.4 mm)*	NA	No pathology	Not applicable	[67]
		Self-aggregated 2N4R tau PFFs (9 µg; 5 µl)			Inclusions	Ipsilateral entorhinal cortex, mammillary area, & contralateral hippocampus	
PS19 (1N4R; P301S; C57BL/6); 2–3 m	1, 3, 6 m PI	Heparin-treated Myc tagged K18 (4R)/P301L tau PFFs (5 µg; 5 µl)	Unilateral; Hippocampus *(A/P* = *-2.5 mm, L* = + *2 mm, D/V* = *-1.8 mm)* Unilateral; Striatum *(A/P* = + *0.2 mm, L* = + *2 mm, D/V* = *-2.6 mm)* Overlaying primary visual cortex *(A/P* = + *0.2 mm, L* = + *2 mm, D/V* = − *0.8 mm)*	NA	Inclusions	Substantia nigra, thalamus, locus coeruleus, dorsal raphe nuclei, & neocortex	[75]
		Heparin-treated Myc tagged 4R2N/P301S tau PFFs (10 µg; 5 µl)				CA2, dentate gyrus, entorhinal cortex, locus coeruleus, substantia nigra, striatum, thalamus, & corpus callosum	
P301L (2N4R; P301L-tau; C57BL/6); 3 m	2, 7, 14 d 1,2 & 3 m PI	Heparin-treated Myc K18 (4R)/P301L tau PFFs (0.05–25 μg; 2–5 µl)	Unilateral; Hippocampus (*A/P* = *-2.5 mm, L* = + *2 mm, D/V* = − *2.4 mm)*	1 µl/min	NFT-like; Th-S positive structures & argyrophilic inclusions	CA3, dentate gyrus, piriform cortex, & hippocampus	[123]
			Unilateral; Frontal cortex area 3; *(A/P* = + *2 mm, L* = + *2 mm, D/V* = − *2.7 mm)*			Amygdala, thalamus, midbrain, & brainstem	
T40PL-GFP (2N4R; GFP tagged P301L; B6C3H/J); 2–3 m	3 m PI	Heparin-treated Alexa Fluor 594-tagged 2N4R/P301L tau PFFs (2 μg; 2.5 μl)	Unilateral; Hippocampus *(A/P* = *-2.5 mm, L* = + *2 mm, D/V* = *2.4 mm)*	NA	NFT-like	Ipsi- & contralateral CA3, dentate gyrus, subiculum, & retrosplenial granular cortex	[58]
rtg4510 (2N4R P301L; C57BL/6 X FVB); 28 d	1 m & 2.5 PI	Heparin-treated hTau (2N4R) short filaments (SFs) (5 μg; 2.5 μl)	Unilateral; Cerebral motor cortex 1 *(A/P* = *− 2.5 mm, M/L* = *2 mm, D/V* = *1 mm)*	0.5 μl/min	Inclusions	No propagation	[164]
PS19 (1N4R; P301S; C57BL/6); 2.5 m	5 m PI	Heparin-treated K18 (4R) tau PFFs (3 µg; 3 µl)	Unilateral; Hippocampus *(A/P* = *− 2.2, L* = *− 1.6, D/V* = *− 1.2)*	0.3 µl/ min	NT-like	Ipsi- & contralateral hippocampus & cerebral cortex	[162]
PS19 (1N4R; P301S; C57BL/6); 3 m	1 & 3 m PI	Heparin-treated His tagged K18 (4R) tau PFFs (5 μg; 5 μl)	Unilateral; Hippocampus *(AP* = − *2.5 mm, L* = − *2.0 mm, DV* = − *1.8 mm)*	0.2 μl/min	Inclusions	Ipsi- & contralateral hippocampus, ipsilateral dentate gyrus, entorhinal cortex, retrosplenial cortex	[173]
		Heparin-treated IAPP-K18 (4R) tau PFFs (5 μg; 5 μl)					
P301L (2N4R, P301L; C57BL/6); 4 m	6 w PI	Heparin-treated His-tagged K18 (4R)/P301L tau PFFs (5 µg; 1 µl)	Unilateral; Hippocampus (*A/P* = *-1.8 mm, L* = *-1.72 mm, D/V* = *-1.8 mm)*	0.2 µl/min	NFT-like	Ipsi- & contralateral hippocampus	[4]
PS19 (1N4R; P301S; C57BL/6); 2–3 m	14 d, 1,3, 6 & 12 m PI	Heparin-treated Myc tagged 2N4R/P301S tau PFFs (4 µg; 1 µl)	Unilateral; Locus Coeruleus *(A/P* = − *5.45 mm, L* = + *1.28 mm, D/V* = − *3.65 mm)*	0.1 µl/min	NFT- & NT-like	Ipsilateral locus coeruleus, nucleus prepositus hypoglossi, nucleus paragigantocellularis, hypothalamus, amygdala, bed nucleus of the stria terminali, frontal cortex, & spinal cord	[76]
PS19 (1N4R; P301S; C57BL/6); 3 m	1.5, 3.5, 6 and 12 m PI	Heparin-treated K18(4R)/P301L tau PFFs (333 μM; 5 µl	Unilateral; Hippocampus *(A/P* = *-2.0 mm, L* = + *1.4 mm, D/V* = *-1.2 mm)* Overlying frontal Cortex *(A/P* = + *2.0 mm, L* = + *1.4 mm, D/V* = − *1.0 mm)*	1 µl/min	NFT-like	Ipsi- & contralateral hippocampus & frontal cortex	[144]
			Entorhinal cortex *(A/P* = − *4.8 mm, L* = − *3.0 mm, D/V* = − *3.7 mm)*			Subiculum, hippocampal formation, amygdala, thalamus & frontal cortex	
			Substantia Negra *(A/P* = − *4.8 mm, angle 16, L* = − *1.1 mm, D/V* = − *4.7 mm)*			Striatum, thalamus, brain stem & cortical regions including the motor cortex	
PS19 (1N4R; P301S; C57BL/6); 4 m	3 m PI	Heparin-treated K18(4R)/P301L tau PFFs (66.7 µM; NA)	Unilateral; Hippocampus *(A/P* = *− 2.0 mm, L* = + *1.4 mm, D/V* = *− 1.4 mm)* Overlying frontal cortex *(A/P* = + *2.0 mm, L* = + *1.4 mm, D/V* = *− 1.0 mm)*	1 µl/min	NFT-like	Ipsi- & contralateral hippocampus & frontal cortex	[155]
		Aβ-induced K18(4R)/P301L tau PFFs (66.7 µM; NA)					

*NA* Not available; *PI* Post inoculation; *T40* Full length tau; *X-Tau* Cofactor-free self-seeding tau; *K18* Truncated tau containg 4 repeats; *IAPP* Islet amyloid polypeptide; *A/P* Anterior/posterior; *L* Lateral; *D/V* Dorsal/ventral

**Table 5:** Summary of studies that involved modulation of tau propagation

Experimental animal; age at inoculation	Time of termination	Pathological tau (mass; volume)	Brain region of isolation	Application; injection region (coordinates from bregma)	Speed of application	Pathology	Propagation	Modulation	References
SHR72 (2N4R, truncated tau aa151–391; SHR); 2 m	4 m PI	Sarkosyl insoluble AD-tau (0.6 & 0.9 µg; 1.5 µl/site)	Parietal cortex	Bilateral; Hippocampus *(A/P* = *− 3.6 mm; L* = + */ − 2.0 mm; D/V* = *3.3 mm)*	1.25 μl/min	NFT-like	Ipsilateral CA1, CA2, CA3	Enriched environment reduced tau pathology	[107]
P301S (1N4R; P301S tau; C57BL/6); 4–4.5 m	2 m PI	Sarkosyl insoluble AD-tau (0.3 µg; 3 µl)	Cerebral cortex	Unilateral; Hippocampus *(A/P* = *-2.5, L* = + *2 mm, D/V* = *-1.8 mm)*	NA	NFT-like	Ipsi- & contralateral hippocampus	Chronic intermittent hypoxia enhanced tau pathology	[86]
PS19 (1N4R; P301S; C57BL/6); 3 m	2 m PI	Myc tagged K18(4R)/P301L tau PFFs (333 μM; 5 μl)	Synthetic	Unilateral; Frontal cortex *(A/P* = + *2.0 mm, L* = + *1.4 mm, D/V* = *− 1.0 mm)*	1 μl/min	NFT-like	Cortex	Inhibition NLRP3–ASC or knock out for ASC reduced tau propagation	[142]
PS19 (1N4R; P301S; C57BL/6); 3 m + MCC950 (Inhibitor of NLRP3–ASC)									
PS19 ASC (1N4R; P301S & ASC + / + ; C57BL/6); 3 m									
PS19 ASC KO (1N4R; P301S & ASC -/-; C57BL/6); 3 m									
PS19 (1N4R; P301S-tau; C57BL/6); 3.5 m	4.5 m PI	K18(4R)/P301L tauu PFFs (333 μM; 5 μl)	Synthetic	Unilateral; Hippocampus *(A/P* =−*2.0 mm, L* = + *1.4 mm, D/V* =−*1.4 mm)* Frontal cortex *(A/P* = + *2.0 mm, L* = + *1.4 mm, D/V* =−*1.0 mm)*	1 μl/min	Inclusions	Ipsilateral hippocampus & frontal cortex	Absence of NLRP3 led to reduction in tau propagation	[143]
PS19 NLRP3 KO (1N4R; P301S-tau & NLRP3 -/-; C57BL/6); 3.5 m									
PS19 NLRP3 (1N4R; P301S-tau & NLRP3 + / + ; C57BL/6); 3.5 m									
PS19 (1N4R; P301S; C57BL/6); 3 m PS19 (1N4R; P301S; C57BL/6); 3 m + colony-stimulating factor 1 receptor inhibitor (PLX5622) PS19, Ikbkb inactivation (1N4R; P301S; Ikbkb -/-, C57BL/6); 3 m PS19, Ikbkb activation (1N4R; P301S; Cx3cr1 CreERT2/ + ; IkbkbCAF/F), 3 m	1 m PI	Brain homogenates from PSP (12.9 μg; 3 μl)	NA	Unilateral; Hippocampus *(A/P* = *− 2.5 mm, L* = + *2 mm, D/V* = * − 1.8 mm)*	NA	Inclusions	Ipsilateral hippocampus & cortex Ipsilateral hippocampus & cortex Ipsilateral cortex Ipsilateral hippocampus	Removal of microglia or inactivation of NF-kB reduced tau propagation in the cortex, activation of NF-kB increase tau propagation in the hippocampus	[159]
		K18 P301L tau (5 μg; 2 μl)	Synthetic						
		K18 P301L tau) (0.4 μg; 2 μl)							
PS19 (1N4R; P301S; C57BL/6);	2 m PI	rTg4510 brain homogenates (NA; 2 μL)	NA	Unilateral; Hippocampus *(A/P* = *− 2.5 mm, L* = *− 2 mm, D/V* = *− 1.8 mm)*	NA	NFT-like	Ipsi- & contralateral hippocampus	Absence of Atg7 enhanced tau pathology	[167]
PS19 Atg7 KO (1N4R; P301S Atg7^fl/fl^ and Cx3cr1^CreER^; C57BL/6); 2–3 m									
5xFAD/PS19 (1N4R; P301S; C57BL/6);4 m	3 m PI	Heparin-treated K18(4R)/P301L tau PFFs (333 µM; 5µL)	Synthetic	Unilateral; Hippocampus *(A/P* = *− 2.0 mm, L* = + *1.4 mm, D/V* = *− 1.4 mm)* Overlying frontal cortex *(A/P* = + *2.0 mm, L* = + *1.4 mm, D/V* = *− 1.0 mm)*	1 µl/min	NFT-like	Ipsi- & contralateral hippocampus & frontal cortex	Depletion of microglia attenuates Aβ‑facilitated tau pathology and neurodegeneration	[103]

*NA* Not available; *KO* Knock out; *KI* Knock in; *PI* Post inoculation; *A/P* Anterior/posterior; *L* Lateral; *D/V* Dorsal/ventral

### Modulation of tau propagation

In recent decades, a large body of clinical evidence on the modulation of tau pathology has surfaced [33, 40, 132]. In this section, we will discuss those factors that can influence tau propagation in inoculation-based models.

The extracellular accumulation of amyloid-beta (Aβ) represents one of the two key pathological features of AD. While, in the early stages of the disease, the distributions of these two distinct lesions do not overlap in terms of neuroanatomy [21, 150], in the later stages, tau pathology has been observed to propagate from the medial temporal lobe to the neocortex specifically in individuals positive for Aβ [152]. The impact of Aβ on tau propagation was explored in two independent studies employing the APP-KI and 5xFAD mouse models. The presence of Aβ plaques was found to facilitate the rapid amplification of inoculated tau-seeds into large tau aggregates, and promote their distal propagation [70, 156]. Tau inoculation in the 5xFAD/PS19 model that mimics ATN pathology, further confirmed these findings [103]; suggesting that Aβ can partially participate in tau propagation.

In addition to the neuro-centric perspective on tauopathies, the investigation of resilience mechanisms has been broadened to encompass the role of protective blood–brain barriers, the vasculature, and, most notably, glia. Microglia, which have been extensively studied in both AD and experimental models of tauopathies [17, 78, 101, 179], play a complex and sometimes contradictory roles in tau pathology. On one hand, microglia can facilitate the removal of tau by internalizing and processing extracellular tau and synapse housing tau aggregates [104]. On the other, under specific conditions, microglia have also observed to propagate pathological tau [73, 145]. To explore this conundrum, various approaches, such as depleting microglia or modifying their function by suppressing the transcription factor nuclear factor kappa-light-chain-enhancer of activated B cells (NF-κB), impeding autophagy, or deactivating inflammasomes have been employed [79, 103, 120, 142, 143, 158, 159].

In tau propagation models, a significant reduction in tau inclusions is observed following the depletion of microglia and subsequent inoculation with either PSP brain extract or synthetic K18/P301L tau fibrils in PS19 mice or 5xFAD/PS19 [103, 159]. NF-κB, a transcription factor implicated in neuroinflammation, regulates a diverse array of genes [56, 91, 118, 121]. Its activation entails a sequence of events: signal detection, activation of the IκB kinase complex, phosphorylation of IκB mediated by IKKβ, degradation of IκB, and the eventual nuclear translocation of NF-κB, enabling gene transcription [174]. In the context of AD, anomalies in NF-κB expression or function have been reported [27, 84, 141, 148]. Notably, by attenuating IKKβ expression and consequently NF-κB levels, a marked decrease in tau inclusions at the ipsilateral side of inoculation was observed, mirroring the effect of microglial depletion [159]. Complementary experiments revealed that an upsurge in IKKβ expression exacerbated pathology, thereby underscoring the pivotal role of microglial NF-κB activation in the spread of tau [159]. This manipulation of microglia offered insightful perspectives into their paradoxical role in tauopathies.

The autophagy-lysosomal pathway plays a vital role in the homeostasis of tau protein [81, 177]. Autophagy related 7 protein (ATG7), essentially serves as a critical facilitator of autophagosome maturation [31]. Deficiencies or alterations in ATG7 expression or function can result in metabolic disruptions and obstruct the uptake and clearance of extracellular tau, thereby potentially exacerbating the pathological accumulation of tau protein [105]. In ATG7 knockout mice, ATG7 deficiency promoted pro-inflammatory response and inflammasome activation. Inoculation of rTg4520 mice brain-derived tau demonstrated that the microglial ATG7 deficiency enhanced intraneuronal tau propagation [168].

The inflammasome is a multiprotein complex that plays a crucial role in the innate immune system [66, 93, 94]. NLRP3 inflammasome, named after its central component, NOD-, LRR- and pyrin domain-containing protein 3 (NLRP3), is one of the best-studied inflammasome and is known to be activated by a wide variety of stimuli. Once activated, NLRP3 interacts with the adaptor protein, apoptosis-associated speck-like protein containing a CARD (ASC), which in turn recruits pro-caspase-1, resulting in the formation of the NLRP3 inflammasome complex [92, 93]. Aggregated tau can activate the NLRP3–ASC inflammasome, contributing to the disease progression [142]. To study the effect of NLRP3-ASC inflammasome on tau propagation, knock-out of either NLRP3 or ASC based PS19 mice were utilized [142]. Either ASC deficiency or its chronic inhibition (using MCC950) reduced tau propagation post inoculation of pre-aggregated K18 tau fibrils [142]. Similarly, NLRP3 knock-out mice demonstrated reduction in tau propagation post inoculation of the same fibrillary aggregates [143].

Hypoxia is another significant factor that contributes to tau propagation [172]. Chronic intermittent hypoxia (CIH), which is predominantly associated with conditions such as obstructive sleep apnoea (OSA) and apnoea of prematurity best reflects the development of cognitive decline and AD in elderly population [98, 169]. Individuals with OSA have been observed to exhibit a more rapid longitudinal increase in the levels of CSF total-tau and phospho-tau, associated with AD [24]. In tau propagation models, following AD-tau inoculation in P301S mice, CIH exposure significantly exacerbated tau pathology and propagation in the mice. Furthermore, CIH treatment also enhanced burden of phospho-tau and activated microglia in both WT and P301S tau mice [86].

Epidemiological research has shown that cognitive stimulation and physical activities can play significant roles in slowing down the progression of AD [18, 41, 130, 136]. In addition, enriched environment (EE) have been shown to bolster neuronal activity and therefore, improve cognitive abilities such as functional outcomes, learning capacity and spatial memory [14, 52, 97]. In SHR72 rats expressing human truncated 4R tau, following AD-tau inoculation, EE was found to reduce the tangle pathology and improve navigation ability [107].

To summarize, it appears that restraining or impairing certain pathways such as NF-κB or NLRP3-ASC inflammasome can contribute to a reduction in tau propagation. Conversely, alterations in autophagy (through ATG7 deficiency) or conditions of hypoxia seem to enhance tau propagation. While the mechanism by which an EE reduces tau pathology is not yet clear, it can be hypothesized that an EE may shift microglia into the alternative phenotypes that play a role in neuroprotection through various molecular mechanisms that affect synaptogenesis, neurogenesis, and neuronal activity, leading to enhanced clearance of tau aggregates. Additionally, higher levels of tau may be secreted into the interstitial fluid due to increased neuronal activity, where it can be taken up and processed by activated microglia. These results suggest that microglia may represent a crucial modifier of the progression of tau propagation.

## Conclusion

Thoughtful modelling approaches for tau propagation have aided the field enormously by providing a human-like testing ground and fuelling progress in understanding tau propagation. Tau propagation models can undeniably recapitulate aggregate transmission and replicate *bona fide* pathological inclusions specific to the utilized tau inoculum.

Tau propagation models have proven to be valuable for investigating the mechanisms of tau propagation, and downstream processes through genetic, pharmacological, and non-pharmacological manipulation. Additionally, these models have been already used in preclinical studies on phospho-tau or conformation-specific antibodies [4, 34, 35, 60, 129, 153].

To summarize, we recommend that future preclinical studies to incorporate the following points:

1. Thoroughly characterize the tau inocula, both biophysically and biochemically
2. Rationalize the site of inoculation based on disease-specific affected areas
3. Account for the choice of model to precisely recapitulate the disease specific pathological inclusions
4. Characterize fibrillary pathology using immunohistochemical and histopathological staining.

We strongly recommend that future studies should not be limited to traditional transgenic tauopathy models but also include inoculation-based tau propagation models to make the data as relevant as possible for the translation of drugs into clinical trials. While models are by definition incomplete and imperfect, the above factors should be taken into consideration. Pending on the research question and particularly pending on the preclinical study (specific tauopathy) to be performed the importance of these different aspects needs to be carefully evaluated and taken into account.

## Abbreviations

AD: Alzheimer’s disease
ARTAG: Aging-related tau astrogliopathy
AGD: Argyrophilic grain disease
AFM: Atomic force microscopy
EM: Electron microscopy
BCA: Bicinchoninic acid assay
CD: Circular dichroism
CBD: Corticobasal degeneration
Cryo-EM: Cryogenic-electron microscopy
ELISA: Enzyme-linked immunosorbent assay
DS: Down syndrome
DSAD: Down syndrome indistinguishable from AD
EE: Enriched environment
FRET: Förster resonance energy transfer
FTLD-17: Frontotemporal dementia and parkinsonism linked to chromosome 17
FPLC: Fast protein liquid chromatography
IHC: Immunohistochemistry
IEM: Immunoelectron microscopy
iPSCs: Induced pseudo-stem cells
GGT: Globular glial tauopathies
NFTs: Neurofibrillary tangles
NP: Neuritic plaques
NTs: Neuropil threads
MCI: Mild cognitive impairment
PART: Primary age related tauopathy
PET: Positron electron tomography
PFFs: Preformed tau fibrils
PiD: Pick’s disease
PTMs: Post-translational modifications
PSP: Progressive supranuclear palsy
SEC: Size-exclusion chromatography
TD: Tangle-only dementia
Tg: Transgenic
TEM: Transmission electron microscopy
WB: Western-blot
WT: Wild-type
